# Language Access is a Catalyst for Early Numerical Abilities

**DOI:** 10.1111/desc.70195

**Published:** 2026-04-27

**Authors:** Stacee Santos, Hiram Brownell, Marie Coppola, Anna Shusterman, Sara Cordes

**Affiliations:** ^1^ Department of Psychology & Neuroscience Boston College Chestnut Hill Massachusetts USA; ^2^ Department of Psychological Sciences & Linguistics University of Connecticut Storrs Connecticut USA; ^3^ Psychology Department Wesleyan University Middletown Connecticut USA

**Keywords:** approximate number system, counting, deaf and hard of hearing, language, spontaneous focus on number

## Abstract

Recent research has found that the cumulative amount of time a child has had auditory access to language accounts for differences in early numerical abilities between oral Deaf and Hard‐of‐Hearing (DHH) and hearing preschoolers. We replicate and extend these findings by exploring the role of language ability and salience of numerical information with a larger sample (*N* = 89; 43 female; majority White) of 3– 6‐year‐old oral DHH and hearing children. We captured number knowledge, numerical discrimination, salience of numerical information, and parent‐reports of vocabulary and language experience. Oral DHH children underperformed on the numerical tasks and vocabulary, however, disparities in numerical measures disappeared when controlling for differences in language experience. The salience of numerical information was similar for both groups.

## 1 Introduction

The vast majority of Deaf and Hard of Hearing (DHH) children are born to parents who do not know a sign language and consequently have no access to language early in development (Mitchell and Karchmer 2004). Most DHH children use listening and spoken language (referred to as ‘oral DHH’ children hereafter) by relying upon hearing technology such as hearing aids or cochlear implants (Dougherty 2017). Accordingly, oral DHH children experience a period of language deprivation that coincides with the amount of time it takes for the child's device to be fit or activated, ranging from a few months to a few years after birth (Boston Children's Hospital 2025). While the impact of this language deprivation on oral DHH children's language abilities has been documented (e.g., Lederberg et al. 2000; Lederberg and Spencer 2009), only recently has research highlighted a less obvious connection between this delayed language exposure in infancy and robust numerical delays found in oral DHH children (Santos et al. 2023).

Decades of research have established that oral DHH children underperform on formal (standardized testing) and informal (counting, number games) assessments of math abilities compared to same‐aged hearing peers (HP; e.g., Hine 1970; Pagliaro and Kritzer 2013; Shusterman et al. 2022; Wollman 1964; Wood, et al. 1984; see Santos and Cordes 2022 for review). Importantly, comparable numerical delays are not present in DHH children born to d/Deaf parents who use a sign language (Walker et al. 2024) as these children are provided access to a complete, natural language from birth. Thus, it seems clear that early language deprivation is a primary contributing factor to numerical delays found in oral DHH children.

Santos et al. (2023) explored the source of the disparities in symbolic (number knowledge) and nonsymbolic (numerical discrimination) numerical abilities between oral DHH preschoolers and their HP by considering the unique role of language experience. To do so, they determined each child's Hearing Age, which was defined as the cumulative amount of time that the child had auditory access to language. In line with prior work, they found oral DHH preschoolers as young as 3‐years‐old demonstrated lower number knowledge and lower numerical discrimination acuity than HP. However, strikingly, group differences in performance disappeared in both tasks when Hearing Age replaced Chronological Age in the analyses. For example, 4‐year‐old oral DHH children who receive amplification at 1 year of age (Hearing Age of 3 years) performed similarly to three‐year‐old HPs, implying that language access may be a catalyst for emerging numerical concepts early in development. Notably, differences in auditory access to language fully accounted for group differences in performance on numerical tasks only; group differences in vocabulary levels persisted even when Hearing Age was considered. Santos et al.’s finding provided a unique glimpse of the source of the challenges oral DHH children endure with their developing numerical concepts.

An important limitation to Santos et al. (2023) was the small sample of oral DHH participants (*n* = 14), limiting the generalizability of these findings. Given the low incidence of deafness (less than 1% of children born with detectable hearing loss in the US each year; Quick Statistics About Hearing, 2024), recruitment of a large sample of DHH children for a single study is challenging. Thus, a primary goal of the current study is to replicate findings of Santos et al. (2023) with a larger sample of oral DHH participants to ensure the robustness of these findings.

Moreover, this study aims to extend prior findings by delineating the exact role language plays in numerical development with a larger sample size. Is it simply exposure to language early in development, or is it the child's own developing language abilities, that scaffolds numerical abilities? The current study gauges the relative impact of language experience (i.e., duration of linguistic input) versus language ability (i.e., vocabulary as an indication of language acquired) on the development of early numerical concepts. Santos et al. (2023) found that the amount of time a child has had access to language was a strong predictor of numerical abilities; however, there is good reason to believe that language abilities can also be a primary driver of numerical abilities. For example, research with hearing children has shown a connection between general vocabulary abilities and number knowledge (Negen and Sarnecka 2012), and that mastery of the verbal count routine appears to align with notable improvements in numerical discrimination acuity (Shusterman et al. 2016). Thus, a second goal of the current study is to compare the relative contributions of language experience and language abilities on preschool numerical abilities, to clarify exactly how language promotes numerical abilities in oral DHH preschoolers.

Lastly, we will provide the first investigation into whether lower numerical abilities in oral DHH preschoolers are explained by differences in their predilection to Spontaneously Focus on Number (SFON; Hannula and Lehtinen 2005) in their environment. While prior research with hearing children has revealed SFON to be shaped by linguistic input (Hannula‐Sormunen et al. 2020), related to number knowledge in preschoolers (Savelkouls et al. 2020), and predictive of later math abilities (Hannula et al. 2010; Luomaniemi et al. 2021), no work has yet explored how SFON in oral DHH children compares to SFON in HP. One possibility is that lower levels of SFON in oral DHH children may mediate the relation between language and numerical abilities in this population; that is, less language access in early childhood may result in lower levels of SFON (due to less overall numerical input), which could contribute to lower numerical abilities in childhood. In the current study, we include two measures of SFON to: (1) provide the first characterization of SFON in oral DHH children, and (2) explore whether differing levels of SFON account for the relation between language experience and numerical abilities.

The purpose of the present study is four‐fold: 1) confirm group differences in numeracy between oral DHH and HP preschoolers with a significantly larger sample, 2) explore the relative contributions of language experience and language abilities to developing numerical abilities, 3) provide the first characterization of SFON in oral DHH children, and 4) explore SFON as a potential mechanism responsible for the relation between language and numerical abilities.

## 2 Methods

### 2.1 Participants

Forty‐five oral DHH children (22 assigned female at birth; *M* = 4.87 years; *SD* = 1.09) and 44 hearing peers (21 assigned female at birth; *M* = 4.5 years; *SD* = 0.87) between 2y9m and 7y1m of age participated in the study. Three additional hearing children were excluded because of computer issues. All children in this study used English as their primary and preferred form of communication. Similar to other research, we did not inquire about the presence of chronic otitis media which may reduce auditory language access for both hearing and DHH children using hearing aids (CDC, 2022). However, the chronic condition is uncommon and unlikely to have impacted our findings.

Oral DHH participants were recruited from five DHH preschools across the United States in suburbs of 4 major cities in the Pacific Northwest, Midwest, and New England that focus on oral and auditory language skills, without the use of sign language. Hearing peers were recruited via four mainstream preschools in the United States from suburbs surrounding a major New England city. Our sample included approximately 20% White, 2% Black; 2% Asian, 2% multiracial, and 3% identified as ‘other’, participants (70% of participants did not respond). Highest level of parent education included one percent of our sample with one parent completing high school, six percent with one parent completing some college, 12% with a college (Bachelors) degree, and 34% with an advanced degree (47% did not respond).

All participants were born to hearing parents. Spoken language (English) was reported as the preferred mode and primary source of communication for all participants. While one participant had parents who reported proficiency in sign language at the time of the child's birth, all other parents responded ‘no’ to having signing proficiency at birth. One family (neither parent proficient in sign) estimated using Americal Sign Language (ASL) 50% of the time. An additional nine families indicated very minimal use of basic ASL throughout the day. No other families reported ASL experience.

Hearing loss ranged from moderate to profound. All but one of the DHH participants had a bilateral hearing loss and 28 were diagnosed with a permanent hearing loss at birth. Nine of the 45 DHH children were diagnosed between 15 and 39 months of age, resulting in prolonged language deprivation and late amplification. All language and hearing loss data were obtained through parent report (see Table 1).

**TABLE 1:** Parent‐report hearing and family history for DHH sample (*N* = 45).

Sidedness		Degree of hearing loss	
Bilateral	44	Mild	1
Amplification		Moderate	5
Hearing aids	17	Moderate‐Severe	6
Cochlear implant	17	Severe	8
Hearing aid + cochlear implant	8	Profound	22
		Not Reported	3
Bone anchored hearing aid	3		
		**Family history**	
		Parents DHH	0

### 2.2 Tasks and Procedures

Parents provided written or online informed consent prior to participation. Participants were tested in a quiet area of their preschool. Each child completed four tasks that were administered in the following order: 1) match‐to‐sample (MTS) task (SFON), 2) Imitation Stamp task (SFON), 3) Numerical Discrimination task, and 4) Give‐N (number knowledge). The two SFON tasks were administered first to avoid priming the children to think about number beforehand. The MTS task and Numerical Discrimination tasks were both computerized tasks given on a 13‐inch MacBook Air. All parents completed the Developmental Vocabulary Assessment for Parents (DVAP; Libertus et al. 2015). The parents of oral DHH children were given an additional questionnaire regarding their child's hearing, language exposure, and use of auditory amplification. All procedures were approved by the Institutional Review Board.

#### 2.2.1 Child Measures

##### 2.2.1.1 Spontaneous Focus on Number (SFON)

Two tasks were used to assess children's SFON: a forced choice MTS and an imitation task.

##### 2.2.1.2 MTS

First, a forced‐choice MTS task (Savelkouls et al. 2020) was employed. Prior work has found test‐retest reliability in children for a similar MTS SFON task to be high (Chan and Mazzocco 2017).

To begin, children were introduced to a target stimulus (an image containing anywhere from 1 to 4 orange 1‐in^2^ squares randomly placed on a white background, 17 × 10 cm) at the center of the screen and told, “We are going to play a matching game. I want you to look at this picture [researcher gestured around the Target image] and remember it. When you are ready, I am going to show you more pictures.” After a brief pause, participants were asked, “Are you ready?” When the child indicated that they were ready, the researcher moved on to the next screen. On this screen, the target image disappeared, and children saw two new choice images side‐by‐side and were asked, “Which picture best matches the one you just saw? This picture [researcher gestured around the image on the left] or this picture [researcher gestured around the image on the right]?” Throughout the task, children were given positive encouragement to continue but were given no feedback on their performance.

Each “picture” contained one, two, three, or four, orange squares randomly placed on a white background. Target images were placed in the center of each screen. The choice screen always contained two images placed side by side on the left and right side of the screen. On Standard trials, the correct match pictured the same number and same sized squares as the target stimulus in a different configuration. The incorrect match contained a different number of squares that were also a different size with a different cumulative area than the target image. Consequently, in the Standard trials, the correct response matched the target image in both number *and* cumulative area, whereas the incorrect response did not match the target image on either of these dimensions.

Interspersed randomly with the Standard trials were Probe trials. Probe trials were similar to Standard trials, except that there was no clear “correct” or “incorrect” match. On Probe trials, one choice image (the Number match) had the *same number* of squares as the target image although the squares had a different cumulative area than the target. The cumulative area ratio (set with smaller cumulative area/set with larger cumulative area) ranged between 0.38 – 0.75. The other choice image (the Area match) had a *different number* of squares than the target image (anywhere from 1 to 4), but the size of the squares was adjusted to match the cumulative area (total amount of orange on the screen) to the target.

The MTS task consisted of a total of 17 trials: five warm‐up Standard trials (with feedback) to ensure children understood the task, followed by six Standard trials randomly mixed with six Probe trials. The experimenter never used any words to imply that number or area were relevant dimensions for matching in the task. There were two dependent measures from the MTS task: Accuracy on Standard Trials, and Proportion of Numerical Matches on Probe Trials. Given that correct performance on Standard trials of this task simply required an ability to match on number OR on cumulative area, Standard trial performance was not necessarily considered a measure of SFON or numerical abilities (in line with Savelkouls et al. 2020). Instead, overall accuracy on Standard trials was interpreted as a measure of how well the child followed the demands of a matching task. However, Probe trial performance required children to decide between a numerical match and an area match. Thus, the proportion of numerical matches selected on Probe trials was used as a measure of the child's SFON—their natural proclivities to match the images based upon number.

##### 2.2.1.3 Imitation Task

A stamping imitation task modeled on Perez and McCrink (2019) was used to capture children's SFON of actions performed—specifically, the quantity of stamping behaviors performed by the experimenter. Performance on this action‐based SFON task has previously been found to be associated with prior math abilities in preschoolers (Elliott et al. 2022).

The stimuli included 3 black outline pictures of a flower without petals, a right facing cheetah without spots, and a left facing dinosaur without spikes on its back, each on 8 × 11‐inch white paper. The experimenter introduced the task by telling the child that they were going to play a stamping game. To begin, the experimenter had one copy of the image, and an identical one was placed in front of the child. The child was asked to “Watch what I do very carefully, then do exactly what I do.” The experimenter then stamped the picture to add 4 petals to the flower, 2 spots on the cheetah, or three spikes on the back of the dinosaur (depending on the picture). The child was then given the stamp and reminded to “Do exactly what I did” on their picture. Once the child stopped stamping, the experimenter asked, “Are you done?” to confirm that the child was finished and moved on to the next trial or task without providing feedback. The order of the three trials was randomized.

Trials were coded as correct if the child produced the exact same number of stamps as the experimenter (1). It was otherwise scored as incorrect (0). Thus, children received an overall score between 0 and 3 on this task.

##### 2.2.1.4 Numerical Discrimination

We used a computer program designed to measure nonsymbolic numerical discrimination acuity (Panamath from Halberda et al. 2008). During each trial, participants were shown two side‐by‐side boxes on a grey background on the computer screen. The right box had an array of blue dots inside a blue perimeter with an image of the character Grover just outside the box to the bottom right. The left box had an array of yellow dots inside a yellow perimeter with an image of the Sesame Street character Big Bird just outside the box to the bottom left. The sizes of the individual dots within each array were heterogeneous. To avoid reliance upon dot size or cumulative area, on a random half of the trials, the cumulative areas of the dots in both boxes were equated and on the other half of trials, the average size of the dots in each array were equated (as per Halberda et al. 2008).

On every trial, dots were briefly displayed (display time 2100 ms) and children were asked, “Who has more dots?” Children were instructed to point to or say which box had a greater number of dots. The number of dots within each array ranged between 5 and 21 dots, with the ratio of the number of dots in the larger array to the smaller array ranging between 1.2 to 2.8. The experimenter sat to the side of the screen and pressed the corresponding button to reflect the child's response. Participants received two practice trials at the beginning of the task during which they were given feedback, followed by 24 test trials. No feedback was given during the test trials. Given the age of the children, and prior work showing percent correct to be an appropriate measure of numerical discrimination in this task (Santos et al. 2023; Shusterman et al. 2016), we used this as our dependent measure of numerical discrimination. Previous work has shown this numerical discrimination task has good inter‐test reliability (DeWind and Brannon 2016; Shusterman et al. 2016).

##### 2.2.1.5 Number Knowledge

Lastly, children participated in the standard Give‐N task (based on Santos et al. 2023; Wynn, 1990) to assess their knowledge of number words. Children were presented ten 2‐inchyellow rubber ducks and a 9‐inch blue circular plastic plate. The experimenter explained the task, “These are my ducks, and this (pointing to the plate) is my pond. I am going to put *one* duck into the pond like this” (experimenter places one duck into the “pond” then places it back with the set). “Can you put *one* duck into the pond?” (if the child puts one duck into the pond), “Is that *one* duck?”. When the child acknowledged one duck, the experimenter removed it from the “pond” and placed it with the other ducks. Then the experimenter asked for the next larger set size: “Can you put *two* ducks into the pond?”. After each time the child placed the requested number of ducks in the pond, the experimenter asked “Is that N ducks?” and then “Can you count to make sure?”, regardless of whether the number of ducks matched the size requested. If the child *correctly* placed N ducks into the pond and *correctly* counted the ducks to confirm the quantity, the experimenter continued the task with N+1 ducks and followed the same procedure for 3, 4, 5, and 6 ducks (in that order, with 6 ducks confirmed twice), or until the child failed to correctly place the number asked. If the child placed an *incorrec*t number of ducks, the experimenter would then ask for N‐1 ducks. This procedure continued until the child got N correct twice, and N+1 incorrect twice, or if the child got 6 correct twice. The final number of ducks correctly placed in the “pond” and confirmed (counted) was considered the child's *number knower‐level*. Thus, a child who correctly placed 3 ducks in the pond consistently but made errors when asked for 4 ducks was considered a 3‐knower. Knower‐level scores were coded between 0 and 6, thus, the maximum knower‐level assigned to any child was 6 for analyses. Typically, a child who can accurately and reliably produce of set of 6 objects or more is considered a Cardinal Principle‐knower (or CP‐knower) and is thought to have mastered the counting procedure. Reliability for the titrated version of the Give‐N task has been established in previous research, revealing 77% test‐retest agreement of knower‐levels and incredibly high reliability (weighted Kappa .87; Marchand et al. 2022).

#### 2.2.2 Parent Questionnaires

##### 2.2.2.1 Vocabulary

Parents (*N_DHH_* = 40; *N_HP_* = 35) completed the Developmental Vocabulary Assessment for Parents (DVAP; Libertus et al. 2015) as a measure of the child's vocabulary abilities. The DVAP is a quick, parent‐administered questionnaire comprised of the first 204 words from Form A of the Peabody Picture Vocabulary Test‐4 (PPVT‐4; Dunn and Dunn 2012). The DVAP is designed to be used with children between the ages 2 – 7 years and found to be reliable and correlate strongly with a child's actual and future PPVT‐4 scores (Libertus et al. 2015).

##### 2.2.2.2 Hearing Questionnaire

Parents of DHH children completed an additional questionnaire to obtain information about their child's hearing history. This questionnaire asked parents to report at what age hearing loss was first detected, degree of hearing loss, sidedness, type, hearing technology, how much exposure to a signed language, family history, and educational services and activities. Parents were asked detailed questions to denote the age at which the child began to utilize hearing technology for speech access and this information was used to compute the child's Hearing Age. Hearing Age was calculated by subtracting the age (in months) the child began accessing speech through hearing technology from the child's chronological age at test (in months). For example, if a child was 40 months at time of test and began wearing hearing aids at 10 months, her Hearing Age would be 40–10 = 30 months. Four of the parents of DHH children did not complete the hearing questionnaire, resulting in a Hearing Age for only 41 of the 45 DHH participants. For the Hearing Peer group, Hearing Age was, by definition, the same as chronological age.

### 2.3 Data Analyses

The following 6 dependent variables were used in analyses: 1) Proportion Correct on Standard trials of the MTS task (measure of matching performance); 2) Proportion numerical matches on MTS task (measure of SFON); 3) Imitation Score (0–3 possible, measure of SFON); 4) Proportion Correct on Numerical Discrimination (measure of numerical discrimination acuity); 5) Knower‐level score (measure of number knowledge); and 6) DVAP Vocabulary score (measure of Language Ability).

To first characterize group differences, we subjected each dependent variable to separate conventional multiple regression analyses (Cohen et al. 2003) with chronological age (because performance on each of these tasks improve with age) and group membership (oral DHH or HP) as predictors entered in the same step. Degrees of freedom differ across analyses because of incomplete data for some comparisons. If group differences were observed in this first model, a parallel analysis was conducted using Hearing Age rather than Chronological Age to explore whether group differences were accounted for by differences in early language experiences. To assess the presence of an Age x Group and Hearing Age x Group interaction we first centered Age and Hearing Age by converting values into *z*‐score form to reduce the multicollinearity (correlation) between the product vector and Group (*r_AGE_* = 0.954, *p* < 0.001; *r_HEARINGAGE_* = 0.88; *p* < 0.001). The product vector representing the interaction was initially included in all analyses. This interaction independent variable was removed from the analysis and from discussion if it was not significant.

In addition to conventional multiple regression analyses, we use Bayesian linear regression models for all continuous variables (conducted in JASP, 2023). Bayesian models provide a different, useful perspective over frequentist analyses (for overviews, see Dienes and Mclatchie 2018; Kruschke and Liddell 2018; Wagenmakers et al. 2018). Bayesian statistics do not use traditional significance cut‐offs (i.e., *p* < 0.05), rather the value of the Bayes Factor (BF) itself is considered evidence or strength for the hypothesis. Bayes Factors can imply how much *more likely* the data are to occur under the one model over another. Bayesian analyses are particularly suited for determining whether nonsignificant results (*p* > 0.05) reflect insensitivity in the data or are supportive of the null hypothesis (Dienes and Mclatchie 2018). This is particularly useful in this study because of the exploration of group, all Bayesian findings align with the results of our frequentist statistics. They are only included to provide greater support for our interpretation.

For these analyses, the notation BF_10_ represents the BF for accepting the *alternate* hypothesis over the null model and BF_01_ represents the BF supporting evidence for accepting the *null* hypothesis over the alternative model. For models with multiple effects (i.e., age, group, or vocabulary), we also report BF_incl_ as evidence for the inclusion, or BF_excl_ (equivalent to 1/BF_incl_) as evidence for the exclusion, of the variable in the model. The general interpretation of the strength of the evidence based on the BF is found in Table 2 (Lee and Wagenmakers 2014; van Doorn et al. 2021; Wagenmakers et al. 2011).

**TABLE 2:** Bayesian factor interpretation.

Evidence group	BF
Evidence group	BF
No evidence	< 1
Anecdotal evidence	1–3
Moderate evidence	3–10
Strong evidence	10–30
Very strong evidence	30–100
Extreme evidence	> 100
	*(adapted from* Lee and Wagenmakers 2014)

All Bayesian analyses used JASP neutral Uniform model priors to assume each model was equally likely with default coefficient priors reflecting the Cauchy priors with widths of 0.707 (which can be interpreted as 50% certainty that the effect is between −0.707 and 0.707 for the two‐sided hypothesis (Bartlett n.d.). Previous literature suggests that default priors be used in numerical cognition unless there is strong prior knowledge for the effect sizes (Faulkenberry et al. 2020).

## 3 Results

The results are organized to address four distinct goals: 1) confirm group differences in early numerical abilities between oral DHH and HP preschoolers with a significantly larger sample, 2) explore the relative contributions of language experience and language abilities to developing numerical abilities, 3) provide the first characterization of SFON in oral DHH children, and 4) explore SFON as a potential mechanism responsible for the relation between language and numerical abilities. See Table 3 for descriptive statistics.

**TABLE 3:** Descriptive statistics means (SD).

	DHH (*N* = 45)	HP (*N* = 44)
Chronological age (months)	58.43 (13.10)	54.19 (10.50)
Hearing age (months)	43.40 (17.00)	54.19 (10.50)
Numerical discrimination (% correct)	71.60 (16.50)	79.22 (17.88)
Number knowledge (knower level)	3.98 (2.42)	5.23 (1.60)
MTS standard (% correct)	59.00 (25)	71 (26)
MTS probe (% correct)	63 (25)	68 (25)
Imitation stamp task (total score)	1.70 (1.00)	1.74 (1.23)
Vocabulary (total score)	62.15 (35.72)	94.27 (39.10)

### 3.1 Exploring Group Differences in Performance Across Tasks

#### 3.1.1 Numerical Discrimination

The regression model with both Chronological Age (*z*‐scores) and Group predicting performance on the numerical discrimination task showed an interaction (*F*(3, 81) = 14.46, *p* < 0.001, *R* ^2^_adj_ = 0.33; *BF_10_* = 83,697.13). The Chronological Age_Z_ (*β =* 0.81, *p* < 0.001; *BF_incl_* = 24,213.38), Group (*β* = −0.33, *p* < 0.001; *BF_excl_* = 37.87) and the Chronological Age_Z_ x Group product vector were significant (*β* = −0.40, *t*(81) = −2.88, *p* = 0.005; *BF_incl_* = 11.52). Thus, similar to Santos et al. (2023), we find significant group differences with lower numerical discrimination acuity in oral DHH children compared to their HP. Analogous product vectors representing interactions between Group x Age_Z_ for all other DVs were tested in all regression analyses reported below. The product variable was never significant. These product variables are not discussed further.

**FIGURE 1:**
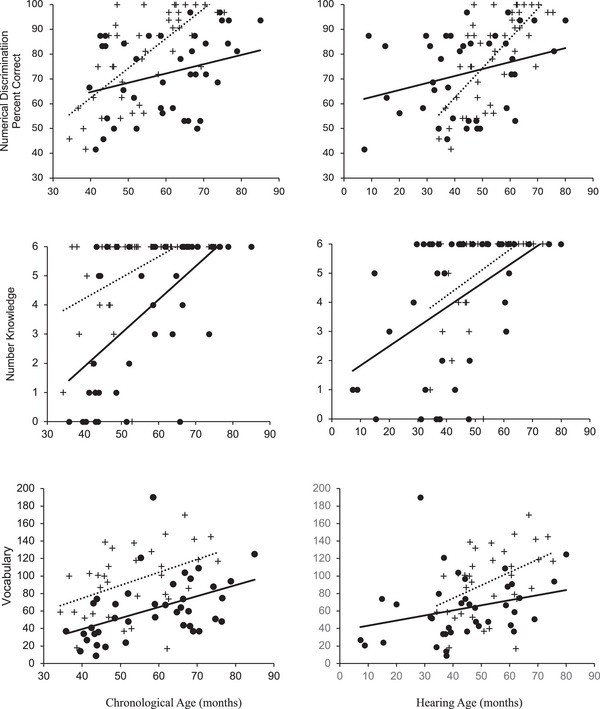
Performance on numerical discrimination, number knowledge, and vocabulary as a function of group and chronological age (on the left) and as a function of group and hearing age (on the right). Crosses represent hearing children's performances, and filled dots represent DHH children's performances. Best fitting regression lines for chronological age and group (dotted line: hearing children and solid lines: DHH children).

However, the significant interaction suggests DHH children show performance increases at a slower rate than HP across the age range (see Figure 1).

When hearing age (*z*‐scores) replaced Chronological Age as a predictor, the overall model was significant (*F*(3, 78) = 13.71, *p* < 0.001, *R* ^2^_adj_ = 0.32; *BF_10_* = 57,451.23). There was very strong evidence to reveal that Hearing Age_Z_ variable was a significant factor in the model (*β =* 1.03, *p* < 0.001; *BF_incl_* = 67,267.48). Importantly, however, there was moderate evidence revealing that Group was not a significant factor (*β* = 0.02, *p* = 0.865; *BF_excl_* = 4.40). Thus, in line with prior work (Santos et al. 2023), we find differences in language experience between groups account for overall differences in performance in the numerical discrimination task. We again found the Hearing Age_Z_ x Group product vector was significant (*β* = −0.64, *t*(78) = −3.64, *p* < 0.001; *BF_incl_* = 71.20) indicating that we find different rates of development in numerical discrimination acuity between the two groups, even when adjusting for differences in language experience (the product vector was also reliable in the model with Hearing Age without centering).

#### 3.1.2 Number Knowledge

The model predicting number knowledge with both Chronological Age and Group was significant, *F*(2, 83) = 25.74, *p* < 0.001, *R* ^2^_adj_ = 0.37; (*BF_10_* = 5.7 × 10 ^+^ ^6^), with Chronological Age (*β =* 0.552, *p* < 0.001; *BF_incl_* = 7,20,732.38) and Group (*β* = −0.390, *p* < 0.001; *BF_incl_* = 859.95) both contributing to the overall model. Replicating prior work, we find oral DHH children to demonstrate lower number knowledge relative to HP of comparable chronological age.

When Hearing Age replaced Chronological Age in the model predicting knower level scores, the model was again significant *F*(2, 80) = 16.13, *p* < 0.001, *R* ^2^_adj_ = 0.27; (*BF_10_* = 13,194.40), with Hearing Age (*β =* 0.488, *p* < 0.001; *BF_incl_* = 9383.11) but *not* Group (*β* = −0.108, *p* = 0.288; *BF_excl_* = 2.97) contributing to the overall model. That is, mirroring prior work (Santos et al. 2023), when accounting for differences in language experience, Bayesian analyses reveal strong evidence that hearing age matters, but anecdotal evidence supporting the claim that group differences are irrelevant. Due to the potential for ceiling effects in our number knowledge variable, a binomial logistic regression was run with ‘all correct’ (aka Cardinal Principle Knower) or ‘not all correct’ (Subset Knower) as the dependent variable. The same pattern of results was observed.

#### 3.1.3 Vocabulary

The model predicting vocabulary scores with Chronological Age and Group was significant, *F*(2, 70) = 17.12, *p* < 0.001, *R* ^2^_adj_ = 0.31; (*BF_10_* = 19,027.27) with both Chronological Age (*β =* 0.420, *p* < 0.001; *BF_incl_* = 324.08) and Group (*β* = −0.480, *p* < 0.001; *BF_incl_* = 2066.78) contributing to the overall model, consistent with group differences in reported vocabulary.

When replacing Chronological age with Hearing Age, the model with Hearing Age and Group was significant, *F*(2, 68) = 10.85, *p* < 0.001, *R* ^2^_adj_ = 0.22; (*BF_10_* = 304.00). However, unlike models predicting the numerical measures, Group (*β* = −0.290, *p* = 0.011; *BF_incl_* = 5.33) continued to be a significant predictor in this model (in addition to Hearing Age, *β =* 0.317, *p* = 0.006; *BF_incl_* = 9.79), with moderate evidence in favor of both factors contributing to the model.

### 3.2 Summary

Results with our much larger sample confirm prior findings (Santos et al. 2023) revealing significant group differences in performance on numerical discrimination, number knowledge, and vocabulary when accounting for chronological age. Oral DHH children performed significantly worse compared to HP across all three measures. When Hearing Age replaced Chronological Age in the models, group differences disappeared for our two numerical measures: numerical discrimination and number knowledge, but *not* vocabulary (identical to that of Santos et al. 2023, see Figure 1). Notably, a significant Group x Chronological Age interaction was found for numerical discrimination suggesting not only group differences in performance, but also differences in the rate of performance increase across the two groups. Notably, when accounting for Hearing Age in the model, the main effect of group disappeared, suggesting differences in language experiences may explain overall group differences in numerical discrimination performance during this age range; however, differences in rates may still persist.

To confirm our findings, in a separate analysis, we matched the groups based upon hearing age (± 1 month), resulting in 21 participants in each group. When analyses were conducted on this smaller sample, we again found no support for group differences in either of the models involving numerical dependent variables (numerical discrimination performance (*p* = 0.53) and number knowledge; *p* = 0.26) but group differences remained in the model predicting vocabulary (*p* < 0.001).

### 3.3 Language Experience Versus Language Ability

Analyses revealed that differences in language experience, as measured by a child's Hearing Age, remove the main effect of group in numerical abilities (numerical discrimination and number knowledge). Not surprisingly, Chronological Age (*r* = 0.326, *p* = 0.005) and Hearing Age (*r* = 0.41, *p* < 0.001) were significantly correlated with Vocabulary, as children who rely on listening and spoken language are only able to acquire new vocabulary once they gain auditory access. Considering prior research has revealed a relationship between vocabulary abilities and developing numerical abilities in hearing children (Negen and Sarnecka 2012; Shusterman et al. 2016), it is possible that group differences in language *abilities* may be the primary driver of group differences in numerical discrimination and number knowledge and not language experience. To explore this possibility, we subjected performance on our numerical discrimination and number knowledge tasks to parallel separate stepwise regression analyses (Cohen et al. 2003) to explore the contributions of Language Ability to performance on these tasks (without accounting for differences in language experience), and also to explore the relative contribution of language ability to group differences above and beyond that already explained by differences in hearing age. Thus, our first model exploring the contributions of language ability alone entered chronological age and group membership (HP or oral DHH) as predictors in the first step, and vocabulary entered into the second step. Then, our parallel secondary stepwise regression exploring the relative contributions of language ability and language experience entered Group and Hearing Age (instead of Chronological Age) as predictors in the first step, before entering vocabulary in the second step.

#### 3.3.1 Numerical Discrimination

##### 3.3.1.1 Language Ability Alone

The first step in the model including Chronological Age, Group, and Age x Group as predictors was significant (*F*(3, 66) = 11.74, *p* < 0.001, *R* ^2^_adj_ = 0.32; *BF_10_* = 6645.35). The Age_Z_ (*β =* 0.81, *p* < 0.001; *BF_incl_* = 24,713.38), Group (*β* = −0.35, *p* < 0.001; *BF_incl_* = 37.87) and the Age_Z_ x Group product vector were significant (*β* = −0.38, *t*(81) = −2.43, *p* = 0.01; *BF_incl_* = 11.52). Note the first step in this analysis differs slightly from that reported earlier because it only includes participants who also had a DVAP (vocabulary) score.

Adding vocabulary to the model in the second step did not significantly improve the model (*F*(4, 65) = 9.23 *p* < 0.001, *R* ^2^_adj_ = 0.33; *BF_10_* = 3642.27). The Age_Z_ (*β =* 0.74, *p* < 0.001; *BF_incl_* = 246.34), Group (*β* = −0.28, *p* = 0.02; *BF_incl_* = 7.31), Age_Z_ x Group product vector (*β* = −0.37, *p* = 0.02; *BF_incl_* = 3.14) continued to be significant, but not Vocabulary (Δ*R* ^2^ = 0.01; *β* = 0.14, *p* = 0.23; *BF_excl_* = 1.43). Thus, our vocabulary measure did not account for significant additional variance in performance on the numerical discrimination task above that already offered by chronological age and group.

##### 3.3.1.2 Language Experience Versus Language Ability

To examine the relative role of language *experience* versus language *ability*, a similar stepwise model was run with Group and Hearing Age (not Chronological Age) predicting Numerical Discrimination performance. The first model was significant, (*F*(3, 65) = 11.56 *p* < 0.001, *R* ^2^_adj_ = 0.32; *BF_10_* = 5576.20). Both Hearing Age_Z_ (*β =* 1.0, *p* < 0.001; *BF_incl_* = 67,267.48) and the Hearing Age_Z_ x Group product vector were again significant (*β* = −0.61, *p* = 0.003; *BF_incl_* = 71.20), but not Group (*β* =−0.03, *p* = 0.80; *BF_excl_* = 4.55), with moderate evidence against including Group.

Adding language ability to the model in the second step did not account for significant additional variance (*F*(4, 64) = 9.30 *p* < 0.001, *R* ^2^_adj_ = 0.33; *BF_10_* = 3867.61). The Hearing Age_Z_ (*β =* 0.91, *p* < 0.001; *BF_incl_* = 697.42) and Hearing Age_Z_ x Group product vector (*β* = −0.56, *p* = 0.01; *BF_incl_* = 13.17) were significant, but not Group (*β* = 0.01, *p* = 0.90; *BF_excl_* = 3.77) or Vocabulary (Δ*R* ^2^ = 0.02; *β* = 0.162, *p* = 0.16; *BF_excl_* = 1.50). Both analyses support the interpretation that vocabulary does not appear to play an important role in numerical discrimination acuity, regardless of language experience.

#### 3.3.2 Number Knowledge

##### 3.3.2.1 Language Ability Alone

The first step in the model with Chronological Age and Group predicting number knowledge was significant, *F*(2, 69) = 20.17, *p* < 0.001, *R* ^2^_adj_ = 0.35 (*BF_10_* = 1,15,820.03), with Chronological Age (*β =* 0.57, *p* < 0.001; *BF_incl_* = 7,20,732.38) and Group (*β* = −0.33, *p* < 0.001; *BF_incl_* = 859.95) both significant predictors.

Language ability did not improve the model or meaningfully account for any additional variance when added in the second step in the model *F*(3, 68) = 14.33, *p* < 0.001, *R* ^2^_adj_ = 0.36 (*BF_10_* = 67,612.90). Age (*β =* 0.50, *p* < 0.001; *BF_incl_* = 1064.92) and Group were important contributors (*β* = −0.25, *p* = 0.03; *BF_incl_* = 5.73), but not vocabulary (Δ*R* ^2^ = 0.02; *β* = 0.17, *p* = 0.16; *BF_excl_* = 1.25, anecdotal evidence). Thus, when group differences were present, vocabulary was not a major predictor of performance on the Give‐N task.

##### 3.3.2.2 Language Experience Versus Language Ability

Notably, when considering the influence of language experience, the first step in the model with Hearing Age and Group predicting Number Knowledge was significant, *F*(2, 67) = 12.41, *p* < 0.001, *R* ^2^_adj_ = 0.25 (*BF_10_* = 858.28). Like previous analyses, Hearing Age (*β =* 0.49, *p* < 0.001; *BF_incl_* = 9383.11) was the only significant predictor in the first step as Group differences disappeared (*β* = 0.07, *p* = 0.53; *BF_excl_* = 1.35).

The second step including Vocabulary was also significant *F*(3, 66) = 10.41, *p* < 0.001, *R* ^2^_adj_ = 0.290 (*BF_10_* = 2001.42) with Hearing Age (*β =* 0.412, *p* < 0.001; *BF_incl_* = 102.73, very strong evidence) *and* vocabulary (Δ*R* ^2^ = 0.051; *β* = 0.260, *p* = 0.03; *BF_incl_* = 2.43, anecdotal evidence) contributing to this model (and not Group (*β* = 0.01, *p* = 0.939; *BF_excl_* = 3.85), showing that once group differences are accounted for by differences in language experience, vocabulary has a similar relation to number knowledge in both oral DHH children and HP.

#### 3.3.3 Summary

Results revealed that language ability was not a significant predictor of numerical discrimination or number knowledge performance when group differences were present (i.e., controlling for Chronological Age). When differences in language experience were controlled in the model, language ability did not prove to be influential for numerical discrimination performance. However, when differences in language experience were held constant in the models, both language experience *and* vocabulary significantly predicted Number Knowledge, suggesting that language ability (i.e., vocabulary) partially accounts for individual differences in number knowledge after group differences are explained.

### 3.4 Characterizing SFON

Next, we characterized performance on our two SFON tasks, exploring whether there were group differences in our SFON measures, and, if so, whether these differences are explained by differences in language experience.

#### 3.4.1 SFON MTS

##### 3.4.1.1 Standard Trials

It is noted that performance on the standard trials was not considered a measure of SFON since (1) feedback was provided on warm‐up trials of this task and (2) correct performance did not necessarily require attention to number alone. However, standard trial performance *was* considered a measure of the child's ability to match images and follow instructions. Children in both groups performed significantly above chance (50%) on the standard trials of the MTS task (*M_DHH_* = 58.7%, *t*(43) = 2.31, *p* = 0.026; *M_HP_* = 70.83%, *t*(43) = 5.23, *p* < 0.001), showing that they were able to match images based upon multiple quantitative cues (both number and size). However, the regression model revealed significant group differences in accuracy on the standard trials with the oral DHH children performing worse than their HP, *F*(2, 85) = 12.59, *p* < 0.001, *R* ^2^_adj_ = 0.21 (*BF_10_* = 780.38), with very strong evidence that Chronological Age (*β =* 0.426, *p* < 0.001; *BF_incl_* = 463.22) and Group (*β* = −0.312, *p* = 0.002; *BF_incl_* = 29.46) both contributing to the overall model.

When accounting for differences in language experience by including Hearing Age instead of Chronological Age in the model, the overall model was significant, *F*(2, 82) = 6.77, *p* = 0.002, *R* ^2^_adj_ = 0.12 (*BF_10_* = 9.32), with strong evidence for Hearing Age (*β =* 0.325, *p* = 0.004; *BF_incl_* = 13.21) but not Group (*β* = −0.104, *p* = 0.348; *BF_excl_* = 1.32, anecdotal evidence) contributing to the overall model, suggesting that early language experience plays a role in group differences in understanding the task demands / matching ability.

##### 3.4.1.2 Probe Trials

**FIGURE 2:**
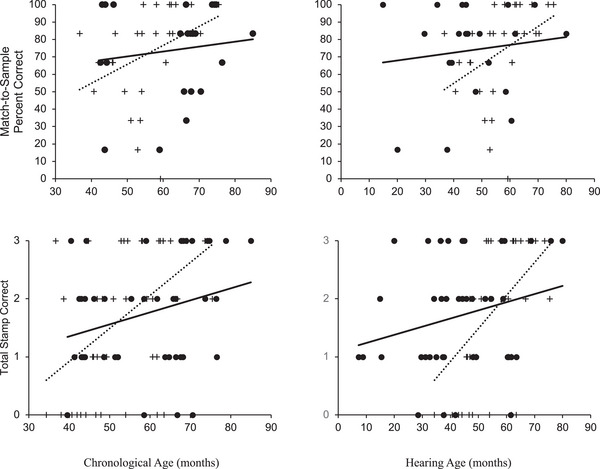
Performance SFON MTS probe and stamp imitation tasks as a function of group and chronological age (on the left) and as a function of group and hearing age (on the right). Crosses represent hearing children's performances, and filled dots represent DHH children's performances. Best fitting regression lines for chronological age and group (dotted line: hearing children and solid lines: DHH children).

Because performance on the standard trials was viewed as indicative of the child's understanding of the task demands, only children that performed above chance (at least 4 of 6, or 67% correct) on the standard trials (*N*_total_ = 52; DHH = 23, HP = 29) were included in the analyses of probe trial performance (when including all children in the model the pattern of results presented here did not change). Notably, on average, both oral DHH and hearing children chose the numerical match on the probe trials significantly more often than the area match (*M_DHH_* = 73.9%, *t*(22) = 4.33, *p* < 0.001; *M_HP_* = 75.9%, *t*(28) = 6.06, *p* < 0.001). However, the model with Chronological Age and Group as predictors of Probe trial performance was not significant, *F*(2, 49) = 2.49, *p* = 0.093, *R* ^2^_adj_ = 0.055 (*BF_01_* = 1.35), and neither variable proved to be a significant predictor (*p*’s > 0.05; *BF_incl_* < 1.7). Thus, our data suggest that SFON, as measured using this MTS task, was comparable in oral DHH children and HP. Thus, performance on this SFON task was likely not impacted by language experience (see Figure 2).

#### 3.4.2 Stamp Imitation

**FIGURE 3:**
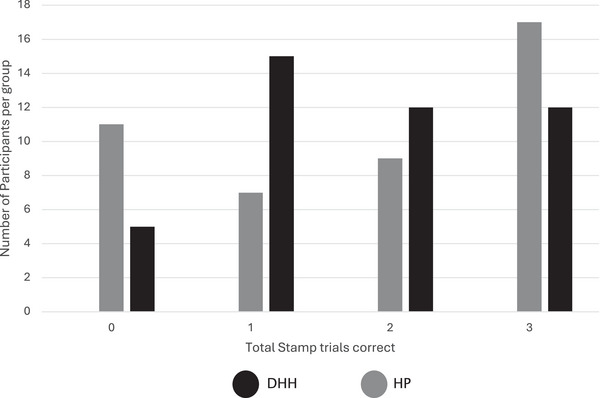
Performance SFON stamp imitation task.

The overall regression model entering Chronological Age and Group as predictors of performance on the Stamp task was significant, *F*(2, 85) = 7.14, *p* = 0.001, *R* ^2^_adj_ = 0.12 (*BF_10_* = 17.30), but Chronological Age (*β =* 0.386, *p* < 0.001; *BF_incl_* = 59.93, very strong evidence) was the only significant predictor of performance. Notably, again, there was moderate evidence that oral DHH children (*M_DHH_* = 1.70, *SD* = 1.00) performed comparably to their HP (*M_HP_* = 1.73, *SD* = 1.23; *β* = −0.085, *p* = 0.406; *BF_excl_* = 3.33) in Stamp Imitation when controlling for chronological age (see Figure 3). Due to the potential for ceiling effects in our stamp imitation task, a binomial logistic regression was run with ‘all correct’ (child produced correct number of stamps for all 3 trials) or ‘not all correct’ (produced an incorrect number of stamps for 1 or more trials) as the dependent variable. The same pattern of results was observed.

### 3.5 Relation Between SFON and Numerical Tasks

Despite significant group differences in performance on numerical tasks (numerical discrimination and number knowledge), our findings surprisingly did not reveal any evidence of group differences in SFON. Nevertheless, as previous research has linked SFON to numerical discrimination acuity (Bull et al. 2014) and number knowledge (Savelkouls et al. 2020), we explored the possibility that differences in SFON may explain the relationship between Hearing Age and Numerical Performance. To do this, our final analyses explored whether performance on either SFON task accounted for group differences in performance on Numerical Discrimination and Number Knowledge tasks. To do so, we ran separate models including Chronological Age, Group, and performance on each SFON task separately predicting performance on our numerical tasks to determine whether SFON performance may account for group differences.

#### 3.5.1 Numerical Discrimination

First, we explored the impact of SFON on numerical discrimination.

##### 3.5.1.1 SFON MTS

We completed a multiple regression predicting performance on our numerical discrimination task with the centered Chronological Age (*z*‐scores), Group, Age_Z_ x Group product vector, and SFON MTS Probe entered in the same step predicting Numerical Discrimination (this analysis only included children scoring above chance on the MTS Standard trials). The overall prediction for this model was strong, *F*(4, 46) = 7.65, *p* < 0.001, *R* ^2^_adj_ = 0.35 (*BF_10_* = 333.07), with Age_Z_ (*β =* .46, *p* = 0.012; *BF_incl_* = 4.75), Group (*β* = −0.38, *p* = 0.004; *BF_incl_* = 15.76), *and* performance on SFON MTS Probe trials (*β =* 0.33, *p* = 0.009; *BF_incl_* = 12.10, strong evidence) contributing to the overall model with no interaction (Age_Z_ x Group *β_AgezxGroup_* = 0.18, *p* = 0.35; *BF_excl_* = 1.82). Critically, although SFON Probe performance was a significant predictor in the model, it is notable that Group differences in numerical discrimination continued to hold.

##### 3.5.1.2 Stamp Imitation Task

We ran comparable analyses using the SFON Stamp Imitation task instead of SFON MTS task as predictors of numerical discrimination. For this model, the overall prediction was strong, *F*(4, 80) = 11.16, *p* < 0.001, *R* ^2^_adj_ = 0.33 (*BF_10_* = 31,316.73), with Age_Z_ (*β =* 0.74, *p* < 0.001; *BF_incl_* = 3512.38), Group (*β* = −0.32, *p* < 0.001; *BF_incl_* = 34.94), Age_Z_ x Group (*β* = −0.38, *p* = 0.01; *BF_incl_* = 10.03) contributing to the overall model but *not* performance on SFON Stamp trials (*β =* 0.11, *p* = 0.28; *BF_excl_* = 2.56, anecdotal evidence).

#### 3.5.2 Number Knowledge

Next, we explored the impact of SFON on number knowledge.

##### 3.5.2.1 SFON MTS Task

A multiple regression analysis including Chronological Age, Group, and MTS Probe in the same step predicting Number Knowledge was strong, *F*(3, 47) = 8.47, *p* < 0.001, *R* ^2^_adj_ = 0.31 (*BF_10_* = 214.32), with Chronological Age (*β =* 0.32, *p* = 0.015; *BF_incl_* = 3.55), Group (*β* = −0.35, *p* = 0.006; *BF_incl_* = 5.54), *and* performance on SFON Probe trials (*β =* 0.33, *p* = 0.01; *BF_incl_* = 7.84, moderate evidence) contributing to the overall model. Again, though SFON Probe performance was related to Number Knowledge, this variable did not account for group differences.

##### 3.5.2.2 Stamp Imitation Task

A comparable analysis was run with the Stamp Imitation task predicting number knowledge. Again, this model was strong, *F*(3, 81) = 15.67, *p* < 0.001, *R* ^2^_adj_ = 0.34 (*BF_10_* = 3,69,444.79), with Chronological Age (*β =* 0.50, *p* < 0.001; *BF_incl_* = 22,971.16) and Group (*β* = −0.37, *p* < 0.001; *BF_incl_* = 407.00) contributing to the overall model, but performance on SFON Stamp trials did not (*β =* 0.114, *p* = 0.235; *BF_excl_* = 2.38, anecdotal evidence).

### 3.6 Summary

Results of our analyses revealed SFON, as measured by the Probe trials of the MTS task, predicted performance on both the numerical discrimination and number knowledge tasks. However, group differences were still significant even when accounting for SFON probe performance, suggesting that differences in SFON do not account for group differences in performance on our numerical tasks. Performance on our Stamp Imitation task did not make a meaningful contribution for either model, suggesting it may be a less robust measure of a child's natural SFON.

## 4 Discussion

There were four primary goals for this study: 1) confirm (with a larger sample) whether differences in early language experience account for group differences in performance on numerical tasks, 2) provide a more thorough exploration of the relative contributions of language experience and language abilities to developing numerical abilities, 3) provide the first assessment of SFON in oral DHH children, and to 4) explore SFON as a potential mechanism responsible for language's impact on numerical abilities.

In line with prior research (Santos et al. 2023), data from this new, larger sample of oral DHH preschoolers revealed considerable evidence to support the claim that a child's language experience (i.e., Hearing Age) is an important contributor to the emergence of early numerical concepts. This is notable as it reveals that language experience early in development, long before the child can produce words, can have a huge impact on later numerical development in preschool, a key precursor to math abilities later in life (Geary 2011). Acknowledging that disparities in performance on numerical tasks historically found between oral DHH and HP are likely a function of disparities in auditory language access, and not differences in aptitude, is valuable when considering how to promote mathematical abilities in oral DHH children.

This study also provided an opportunity for a thorough assessment of the nature of the relationship between language and math learning in preschool. While prior work has found early language experience to fully account for group differences in numerical abilities (Santos et al. 2023), the sample size of that study was small, limiting the generalizability of claims about whether group differences in language abilities may also factor into the equation. While vocabulary abilities are necessarily dependent upon language experience, sometimes greater language input does not equate to greater vocabulary abilities (e.g., Hannula‐Sormunen et al. 2020), as seen here. There are other factors such as differences in the quality of caregiver input (i.e., conversational turns), that may also contribute to individual differences in vocabulary acquisition in the early preschool years. In the current study, we explored the possibility that variations in language ability (i.e., vocabulary size) plays a prominent role in explaining group differences in numerical discrimination and number knowledge performance. Our analyses consistently revealed Hearing Age, but *not* vocabulary, accounted for group differences. This highlights the power of early language access, not language ability, to explain group differences in numeracy.

The results here highlight that the quality and quantity of early language access may be significantly more important than overall vocabulary abilities for emerging numerical concepts. However, when group differences in numerical abilities were removed (by accounting for differences in language access), vocabulary abilities predicted number knowledge, but not numerical discrimination abilities, aligning with prior work (Negen and Sarnecka 2012). This shows that the development of numerical abilities in oral DHH children follows a similar pattern to HP. Finding that overall vocabulary is related to number word knowledge in populations with a very different trajectory of vocabulary and number word learning suggests that this relationship is quite robust.

It should be noted that these results do not align with those of Shusterman et al. (2022). In their study, Shusterman et al. reported child vocabulary size fully mediated group differences in number knowledge in their sample of oral DHH and hearing preschoolers, whereas we did not find this to be the case. It is possible these differences could reflect different measurement tools (behavioral versus parent‐report) used to capture vocabulary ability. Though our parent‐reported vocabulary measure did correlate with number knowledge—consistent with prior work (Shusterman et al. 2016)—it may be that our parent‐reported vocabulary measure was a less sensitive measure of nuances in a child's vocabulary. Future work should consider how different language ability measures factor into the emergence of numerical abilities.

Our results also provide a first look at SFON in DHH children. It was surprising that no group differences were found for either of the SFON tasks, given that prior work shows that SFON is malleable based on numerical language input (Braham et al. 2018). Previous research has shown that children exposed to greater language input influenced SFON (Hannula‐Sormunen et al. 2020). That hearing status may not have a differential influence on a child's spontaneous attention to number is remarkable considering differences in language access (and presumably input) for these two populations. An important next step should expand this work and explore whether number talk in the homes and schools of oral DHH children compare to that of HP (though see Kritzer 2008, 2009). This may help explain how linguistic environments contribute to the development of SFON.

Further, this study corroborates previous research showing that SFON, specifically as measured by the MTS Probe trials, is related to number knowledge (Savelkouls et al. 2021). Though, little research has explored the connection between SFON and numerical discrimination, our findings suggest that a heightened attention to numerical information is also related to refined numerical discrimination acuity. Future research should explore the robustness of this relationship.

These results beg the question, when does early input not matter anymore? Does language experience explain numerical abilities later in development? Future research should explore the reach of the influence language access has on abilities in mathematics in older children. Early numerical concepts are foundational for numeracy. Our findings suggest early identification of DHH populations, coupled with targeted interventions that prioritize language access early in development for oral DHH children are key to closing the gap in math performance, well before elementary school. Understanding the source of the observed challenges oral DHH children face with early number abilities provides unique insight into the connection between language and numeracy. Our results emphasize a different perspective on how language access is critical to the development of number abilities in children.

## Author Contributions

S.S and S.C conceived and designed the study. S.S. collected the data. H.B. contributed analysis tools. S.S., S.C., and H.B. performed the analyses. S.S. and S.C. wrote the paper. All authors contributed to the interpretation of the data, writing the manuscript, and approved the final manuscript.

## Funding

This research was supported by National Science Foundation grants to **Sara Cordes** (NSF#941002), **Marie Coppola** (NSF#1553589), and **Anna Shusterman** (NSF##0845966, #1420966, and #2010547).

## Conflicts of Interest

All authors declare no conflicts of interest.

## Consent

No artificial intelligence assisted technologies were used in this research or the creation of this article.

## Ethics Statement

This research received approval from Boston College IRB.

## Data Availability

The datasets generated during and/or analyzed during the current study are deposited in the Open Science Framework repository and are available from the corresponding author upon reasonable request.

## References

[desc70195-bib-0004] Bartlett, J. (n.d.). An Introduction to JASP: A Free and User‐Friendly …, Study notes Statistics | Docsity. Retrieved December 14 2022. from https://www.docsity.com/en/an‐introduction‐to‐jasp‐a‐free‐and‐user‐friendly/8999270/.

[desc70195-bib-0044] Boston Children's Hospital . 2025. What are Cochlear Implants? https://www.childrenshospital.org/conditions‐treatments/cochlear‐implants.

[desc70195-bib-0005] Braham, E. J. , M. E. Libertus , and K. McCrink . 2018. “Children's Spontaneous Focus on Number Before and after Guided Parent‐Child Interactions in a Children's Museum.” Developmental Psychology 54, no. 8: 1492–1498. 10.1037/dev0000534. 30047774 PMC6132254

[desc70195-bib-0006] Bull, R. , K. Poon , K. Lee , K. Cheah , and M. Mochtar . 2014. Examining Kindergarten Approximation Skills as a Predictor of Children Requiring Learning Support for Mathematics. https://www.academia.edu/93371312/Examining_kindergarten_approximation_skills_as_a_predictor_of_children_requiring_learning_support_for_mathematics.

[desc70195-bib-0050] Chan, J. Y.‐C. , and M. M. M. Mazzocco . 2017. “Competing Features Influence Children's Attention to Number.” Journal of Experimental Child Psychology 156: 62–81. 10.1016/j.jecp.2016.11.008. 28039750

[desc70195-bib-0007] Cohen, J. , P. Cohen , S. G. West , and L. S. Aiken . 2003. Applied Multiple Regression/Correlation Analysis for the Behavioral Sciences, 3rd ed (xxviii, 703). Lawrence Erlbaum Associates Publishers.

[desc70195-bib-0008] Dienes, Z. , and N. Mclatchie . 2018. “Four Reasons to Prefer Bayesian Analyses Over Significance Testing.” Psychonomic Bulletin & Review 25, no. 1: 207–218. 10.3758/s13423-017-1266-z. 28353065 PMC5862925

[desc70195-bib-0048] DeWind, N. K. , and E. M. Brannon . 2016. “Significant Inter‐test Reliability Across Approximate Number System Assessments.” Frontiers in Psychology 7: 310. 10.3389/fpsyg.2016.00310. 27014126 PMC4781867

[desc70195-bib-0009] Dougherty, E. 2017. Studying Language Acquisition in Deaf Children | the Brink. Boston University. https://www.bu.edu/articles/2017/asl‐language‐acquisition/.

[desc70195-bib-0010] Dunn, L. M. , and D. M. Dunn . 2012. Peabody Picture Vocabulary Test—Fourth Edition. 10.1037/t15144-000.

[desc70195-bib-0001] Ear Infections in Children, Babies & Toddlers | NIDCD . (2022). https://www.nidcd.nih.gov/health/ear‐infections‐children.

[desc70195-bib-0051] Elliott, L. , A. M. Silver , A. Imbeah , and M. Libertus . 2022. “Actions May Speak Louder Than Words: Comparing Methods of Assessing Children's Spontaneous Focusing on Number.” Journal of Experimental Child Psychology 214: 105301. 10.1016/j.jecp.2021.105301. 34583264

[desc70195-bib-0011] Faulkenberry, T. J. , A. Ly , and E.‐J. Wagenmakers . 2020. “Bayesian Inference in Numerical Cognition: a Tutorial Using JASP.” Journal of Numerical Cognition 6, no. 2: 231–259. 10.31234/osf.io/vg9pw.

[desc70195-bib-0012] Geary, D. C. 2011. “Consequences, Characteristics, and Causes of Mathematical Learning Disabilities and Persistent Low Achievement in Mathematics.” Journal of Developmental & Behavioral Pediatrics 32, no. 3: 250–263. 10.1097/DBP.0b013e318209edef. 21285895 PMC3131082

[desc70195-bib-0014] Halberda, J. , M. M. M. Mazzocco , and L. Feigenson . 2008. “Individual Differences in Non‐Verbal Number Acuity Correlate With Maths Achievement.” Nature 455, no. 7213: 665–668. 10.1038/nature07246. 18776888

[desc70195-bib-0015] Hannula, M. M. , and E. Lehtinen . 2005. “Spontaneous Focusing on Numerosity and Mathematical Skills of Young Children.” Learning and Instruction 15, no. 3: 237–256. 10.1016/j.learninstruc.2005.04.005.

[desc70195-bib-0016] Hannula, M. M. , J. Lepola , and E. Lehtinen . 2010. “Spontaneous Focusing on Numerosity as a Domain‐Specific Predictor of Arithmetical Skills.” Journal of Experimental Child Psychology 107, no. 4: 394–406. 10.1016/j.jecp.2010.06.004. 20643417

[desc70195-bib-0017] Hannula‐Sormunen, M. , C. Nanu , K. Luomaniemi , et al. 2020. “Promoting Spontaneous Focusing on Numerosity and Cardinality‐Related Skills at Day Care With One, Two, How Many and Count, How Many Programs.” Mathematical Thinking and Learning 22, no. 4: 312–331. 10.1080/10986065.2020.1818470.

[desc70195-bib-0018] Klibanoff, R. S. , S. C. Levine , J. Huttenlocher , M. Vasilyeva , and L. V. Hedges . 2006. “Preschool Children's Mathematical Knowledge: The Effect of Teacher “Math Talk”.” Developmental Psychology 42, no. 1: 59–69. 10.1037/0012-1649.42.1.59. 16420118

[desc70195-bib-0052] Kritzer, K. L. 2008. “Family Mediation of Mathematically Based Concepts While Engaged in a Problem‐solving Activity With Their Young Deaf Children.” Journal of Deaf Studies and Deaf Education 13, no. 4: 503–517. 10.1093/deafed/enn007. 18344538

[desc70195-bib-0053] Kritzer, K. 2009. “Families with Young Deaf Children and the Mediation of Mathematically Based Concepts Within a Naturalistic Environment.” American Annals of the Deaf 153, no. 5: 474–483. 10.1353/aad.0.0067. 19350955

[desc70195-bib-0019] Kruschke, J. K. , and T. M. Liddell . 2018. “The Bayesian New Statistics: Hypothesis Testing, Estimation, Meta‐Analysis, and Power Analysis From a Bayesian Perspective.” Psychonomic Bulletin & Review 25, no. 1: 178–206. 10.3758/s13423-016-1221-4. 28176294

[desc70195-bib-0020] Lederberg, A. R. , A. K. Prezbindowski , and P. E. Spencer . 2000. “Word‐Learning Skills of Deaf Preschoolers: The Development of Novel Mapping and Rapid Word‐Learning Strategies.” Child Development 71, no. 6: 1571–1585. 10.1111/1467-8624.00249. 11194257

[desc70195-bib-0021] Lederberg, A. R. , and P. E. Spencer . 2009. “Word‐Learning Abilities in Deaf and Hard‐of‐Hearing Preschoolers: Effect of Lexicon Size and Language Modality.” Journal of Deaf Studies and Deaf Education 14, no. 1: 44–62. 10.1093/deafed/enn021. 18495655

[desc70195-bib-0022] Lee, M. D. , and E.‐J. Wagenmakers . 2014. Bayesian Cognitive Modeling: a Practical Course. Cambridge University Press. 10.1017/CBO9781139087759.

[desc70195-bib-0025] Libertus, M. E. , D. Odic , L. Feigenson , and J. Halberda . 2015. “A Developmental Vocabulary Assessment for Parents (DVAP): Validating Parental Report of Vocabulary Size in 2‐ to 7‐Year‐Old Children.” Journal of Cognition and Development 16, no. 3: 442–454. 10.1080/15248372.2013.835312.

[desc70195-bib-0026] Luomaniemi, K. , A. Mattinen , J. McMullen , A. Sorariutta , and M. Hannula‐Sormunen . 2021. “The Effects of a SFON‐Based Early Numeracy Program on Multilingual Children's Early Numeracy and Oral Language Skills.” Journal of Cognitive Education and Psychology 20, no. 2: 138–160. 10.1891/JCEP-D-20-00006.

[desc70195-bib-0027] Marchand, E. , J. T. Lovelett , K. Kendro , and D. Barner . 2022. “Assessing the Knower‐Level Framework: How Reliable Is the Give‐a‐Number Task?” Cognition 222: 104998. 10.1016/j.cognition.2021.104998. 35144098

[desc70195-bib-0029] Mitchell, R. E. , and M. A. Karchmer . 2004. “Chasing the Mythical Ten Percent: Parental Hearing Status of Deaf and Hard of Hearing Students in the United States.” Sign Language Studies 4, no. 2: 138–163. 10.1353/sls.2004.0005.

[desc70195-bib-0030] Negen, J. , and B. W. Sarnecka . 2012. “Number‐Concept Acquisition and General Vocabulary Development: General Vocabulary Development.” Child Development 83, no. 6: 2019–2027. 10.1111/j.1467-8624.2012.01815.x. 22803603 PMC3488126

[desc70195-bib-0031] Pagliaro, C. M. , and K. L. Kritzer . 2013. “The Math Gap: A Description of the Mathematics Performance of Preschool‐Aged Deaf/Hard‐of‐Hearing Children.” Journal of Deaf Studies and Deaf Education 18, no. 2: 139–160. 10.1093/deafed/ens070. 23307889

[desc70195-bib-0032] Perez, J. , and K. McCrink . 2019. “Measuring Spontaneous Focus On Space in Preschool Children.” Frontiers in Psychology 10: 2624. 10.3389/fpsyg.2019.02624. 31849753 PMC6892949

[desc70195-bib-0002] Quick Statistics About Hearing, Balance, & Dizziness | NIDCD, 2024. https://www.nidcd.nih.gov/health/statistics/quick‐statistics‐hearing.

[desc70195-bib-0033] Santos, S. , H. Brownell , M. Coppola , A. Shusterman , and S. Cordes . 2023. “Language Experience Matters for the Emergence of Early Numerical Concepts.” NPJ Science of Learning 8, no. 1: 57. 10.1038/s41539-023-00202-w. 38071222 PMC10710413

[desc70195-bib-0047] Santos, S. , and S. Cordes . 2022. “Math Abilities in Deaf and Hard of Hearing Children: the Role of Language in Developing Number Concepts.” Psychological Review 129, no. 1: 199–211. 10.1037/rev0000303. 34138618

[desc70195-bib-0035] Savelkouls, S. , M. A. Hurst , and S. Cordes . 2020. “Preschoolers' Number Knowledge Relates to Spontaneous Focusing On Number for Small, but Not Large, Sets.” Developmental Psychology 56, no. 10: 1879–1893. 10.1037/dev0001099. 32790440

[desc70195-bib-0036] Shusterman, A. , R. Peretz‐Lange , T. Berkowitz , and E. Carrigan . 2022. “The Development of Early Numeracy in Deaf and Hard of Hearing Children Acquiring Spoken Language.” Child Development 93, no. 5: e468–e483. 10.1111/cdev.13793. 35726698

[desc70195-bib-0037] Shusterman, A. , E. Slusser , J. Halberda , and D. Odic . 2016. “Acquisition of the Cardinal Principle Coincides With Improvement in Approximate Number System Acuity in Preschoolers.” PLoS ONE 11, no. 4: e0153072. 10.1371/journal.pone.0153072. 27078257 PMC4831828

[desc70195-bib-0038] Team, J. 2023. JASP (Version 0.18.1) [Computer software].

[desc70195-bib-0039] van Doorn, J. , D. van den Bergh , U. Böhm , et al. 2021. “The JASP Guidelines for Conducting and Reporting a Bayesian Analysis.” Psychonomic Bulletin & Review 28, no. 3: 813–826. 10.3758/s13423-020-01798-5. 33037582 PMC8219590

[desc70195-bib-0040] Wagenmakers, E.‐J. , M. Marsman , T. Jamil , et al. 2018. “Bayesian Inference for Psychology. Part I: Theoretical Advantages and Practical Ramifications.” Psychonomic Bulletin & Review 25, no. 1: 35–57. 10.3758/s13423-017-1343-3. 28779455 PMC5862936

[desc70195-bib-0041] Wagenmakers, E.‐J. , R. Wetzels , D. Borsboom , and H. L. J. van der Maas . 2011. “Why Psychologists Must Change the Way They Analyze Their Data: the Case of Psi: Comment on Bem (2011).” Journal of Personality and Social Psychology 100, no. 3: 426–432. 10.1037/a0022790. 21280965

[desc70195-bib-0042] Walker, K. , E. Carrigan , and M. Coppola . 2024. “Early Access to Language Supports Number Mapping Skills in Deaf Children.” The Journal of Deaf Studies and Deaf Education 29, no. 1: 1–18. 10.1093/deafed/enad045. 38124681

[desc70195-bib-0046] Wood, H. A. , D. J. Wood , M. C. Kingsmill , J. R. French , and S. P. Howarth . 1984. “The Mathematical Achievements of Deaf Children From Different Educational Environments.” The British Journal of Educational Psychology 54, no. 3: 254–264. 10.1111/j.2044-8279.1984.tb02589.x. 6508996

[desc70195-bib-0045] Wollman, D. C. 1964. “The Attainments in English and Arithmetic of Secondary School Pupils With Impaired Hearing.” British Journal of Educational Psychology 34, no. 3: 268–274. 10.1111/j.2044-8279.1964.tb00636.x.

[desc70195-bib-0043] Wynn, K. 1990. “Children's Understanding of Counting.” Cognition 36, no. 2: 155–193. 10.1016/0010-0277(90)90003-3. 2225756

